# A Study of Ziegler–Natta Propylene Polymerization Catalysts by Spectroscopic Methods

**DOI:** 10.3390/ma10050496

**Published:** 2017-05-03

**Authors:** Olga P. Tkachenko, Alexey V. Kucherov, Leonid M. Kustov, Ville Virkkunen, Timo Leinonen, Peter Denifl

**Affiliations:** 1 Zelinsky Institute of Organic Chemistry, Russian Academy of Sciences, Leninsky Prosp. 47, Moscow 119991, Russia; ot@ioc.ac.ru (O.P.T.); akuchero2004@yahoo.com (A.V.K.); 2 National Science and Technology University MISiS, Leninsky prospekt 4, Moscow 119071, Russia; 3 Borealis Polymers Oy, P.O. Box 330, Porvoo F1-06101, Finland; Ville.Virkkunen@borealisgroup.com (V.V.); Timo.Leinonen@borealisgroup.com (T.L.); 4 Borealis Polyolefine GmbH, St. Peter-Strasse 25, Linz A-4021, Austria; Peter.Denifl@borealisgroup.com

**Keywords:** propylene polymerization catalysts, Fourier-transform IR spectroscopy, X-ray photoelectron spectroscopy, electron-spin resonance, XAS

## Abstract

Ziegler–Natta polymerization catalysts were characterized by a complex of surface- and bulk-sensitive methods (DRIFTS, XPS, ESR, and XAS = XANES + EXAFS). A diffuse-reflectance Fourier-transform IR spectroscopy (DRIFTS) study showed the presence of strong Lewis acid sites in different concentrations and absence of strong basic sites in the polymerization catalysts. X-ray photoelectron spectroscopy (XPS), electron-spin resonance (ESR), and (X-ray absorption near-edge structure (XANES) analysis revealed the presence of Ti^4+^, Ti^3+^, Ti^2+^, and Ti^1+^ species in the surface layers and in the bulk of catalysts. The samples under study differ drastically in terms of the number of ESR-visible paramagnetic sites. The EXAFS study shows the presence of a Cl atom as a nearest neighbor of the absorbing Ti atom.

## 1. Introduction

Solid olefin polymerization catalysts represent a very important class of materials. Information about the nature of active sites and spectroscopic information about the Ziegler–Natta polymerization catalysts are scarce. Far more information is available about the kinetics, preparation, and chemistry of the catalysts, including the mechanism of activation of the catalysts by aluminum-containing organic agents and the use of internal donors. Mixed oxides under study represent hybrid materials, which are known to be important for catalytic applications [1]. Several groups have demonstrated various techniques to unravel the state of titanium active species in these catalysts. Spectroscopic investigation of the TiCl_4_–MgCl_2_ systems was done [2]. Carbon monoxide was used as a probe molecule to test the surface sites in the MgCl_2_ support and in the TiCl_4_–MgCl_2_ catalysts of ethylene polymerization. It has been shown that a few types of Lewis acid sites (low-coordinated Mg ions) are present at the surface of the carrier, but they disappear after deposition of TiCl_4_ interacting with the surface sites of MgCl_2_.

The organic component of the Ziegler–Natta catalysts, i.e., internal donors at the surface of the Ti–Mg stereospecific propylene polymerization catalysts, was also studied by diffuse-reflectance Fourier-transform IR spectroscopy (DRIFT) [3]. Ethyl benzoate (EB) and di-n-butyl phthalate (DBP) were chosen as internal donors (ID). These molecules demonstrated a superposition band in the carbonyl region of the IR spectra that can be ascribed to perturbed ID molecules. Deconvolution of this band allowed the authors to distinguish three types of ID complexes in the case of EB; only one of these three was formed in the case of DBP. The data obtained were interpreted from the standpoint of the competition between ID and TiCl_4_ for the surface sites.

A model Ziegler–Natta catalyst on a Pd(111) substrate coated with the MgCl_2_(001) layer was prepared in an ultra-high vacuum and studied using the electron-spin resonance (ESR) technique from the point of view of the formation of radical species during the ethylene polymerization process [4]. Particular attention was paid to the role of AlR_3_ moieties in the reaction, for example, via the interaction with TiCl_4_:

TiCl_4_ + AlR_3_ → RTiCl_3_ → TiCl_3_ + R

The authors also studied the effect of the so-called colored sites (defect sites, F-centers) in the development of the most effective catalyst. The formation of methyl radicals from AlMe_3_ was also detected by the low-temperature ESR spectroscopy. Furthermore, ethyl radicals were detected as a result of the reaction of methyl radicals with AlMe_3_. However, the authors, surprisingly, did not find any Ti^3+^ species in the ESR spectra.

One more type of model Ziegler–Natta polymerization catalysts was prepared by spin-coating on a flat SiO_2_/Si(100) support [5]. XPS analysis of such a catalyst showed the presence of Ti^3+^ species after the thermal treatment of the system at 723 K, when a significant part of the chlorine is removed. 

Yet another approach to study the Ziegler–Natta polymerization catalysts was reported [6]. The use of high-resolution electron microscopy in the investigation of the ethylene polymerization catalysts allowed the authors to discriminate the α-TiCl_3_ phase, which was destroyed rapidly after exposure to ambient air. It should be noted, in view of our further discussion, that such a phase would not reveal any Ti^3+^ paramagnetic centers visible in ESR.

Quite a detailed study of the model Mg–Ti catalysts prepared by gas phase deposition of the components onto the Au polycrystalline foil under UHV conditions (pressures around 1.3 × 10^−5^ Pa) is reported in [7]. The authors found the formation of a TiCl_2_ layer onto the TiCl_4_ layer. Addition of AlEt_3_ into the system leads to the formation of mixed TiR*_n_*Cl_4−*n*_ complexes. Again, no sign of the presence of TiCl_3_ was found in the spectra. These model catalysts were tested in ethylene and propylene polymerization. The fraction of Ti^4+^ species in the total Ti content after the treatment with AlEt_3_ decreased gradually from 0.6 to 0.2 when increasing the AlEt_3_ exposure (measured in Langmuir units).

There are a few papers in which studies were carried out using not model, but real olefin polymerization catalysts [8,9]. The authors investigated the interaction between the EB/TiCl_4_/MgCl_2_ catalyst and an AlR_3_ co-catalyst with the goal of finding a relationship between the degree of titanium reduction and the activity in the olefin polymerization reaction. They observed the dependence of the Ti^2+^ and Ti^3+^ contents determined by redox titration on the Al/Ti ratio, activation temperature, and aging time or reaction time. For instance, for the catalytic systems activated at 70 °C for 2 h at the Al/Ti ratio of 200, the following distribution was revealed from the XPS data: Ti^4+^:Ti^3+^:Ti^2+^ = 9:31:60, i.e., even after severe reduction conditions, a portion of titanium remains in the non-reduced state. The conclusion has been drawn that for ethylene polymerization both Ti^3+^ and Ti^2+^ species seem to contribute to the overall activity, whereas in the case of propylene conversion the major contribution is likely made by the higher oxidation states (Ti^4+^ and Ti^3+^), with the latter being most active in the title process. Similar conclusions were made in the second paper of the Italian team [9]. 

The Japanese group [10,11] reported on the interaction of the TiCl_4_/MgCl_2_ catalyst with an internal donor and found that the position of the Ti XPS peak does not change when varying the nature of the internal donor and the preparation method. The peak position was, however, different for a model TiCl_4_-ester complex, which indicated that such a complex is not formed at the surface, and titanium most probably was present in the reduced state. Thus, the authors concluded that the internal donor could only influence the state of titanium via the intermediacy of MgCl_2_. 

ESR spectroscopy is an informative method in the physicochemical study of Ti catalysts for olefin polymerization. However, in spite of the frequent use of ESR in monitoring the formation and role of paramagnetic sites in polymerization reactions, a lot of conflicting data are reported about the nature of catalytically active sites and active species formed [12,13,14,15,16,17,18,19,20]. Both the influence of the Ti oxidation state on the catalyst properties and the catalytic activity of cationic Ti^3+^ complexes are mainly claimed [12,13,17,18]. In a study of the copolymerization of styrene and ethylene [14], ESR spectroscopic analysis combined with copolymerization kinetics results suggested the presence of a Ti^4+^ active center that is responsible for the formation of polyethylene, a Ti^3+^ species active in the syndiospecific polymerization of styrene, and, moreover, the presence of a third intermediate contributing to the promotion of the copolymerization of styrene with ethylene. The formation of paramagnetic Ti_2_^7+^ pairs is discussed [8]. In the ESR study of the Cp*TiMe_3_/B(C_6_F_5_)_3_ system, it was shown that a small portion (<0.01%) of titanium is occasionally present during polymerization as a complex of Ti^3+^, thereby suggesting that a contribution of the Ti^3+^ species to the catalytic process is unlikely. Different Ti^4+^ species are identified using ^13^C- and ^1^H-NMR spectroscopy, and the portion of ESR-active Ti^3+^ species in the Cp*TiCl_3_/MAO system is evaluated as <1% [9].

Thus, most studies published so far are related to the model catalysts and only the XPS method was used in studying the Ziegler–Natta polymerization catalysts. In this paper, we present details of characterization of the Ziegler–Natta polymerization catalysts by a complex of surface- and bulk-sensitive methods (DRIFTS, XPS, ESR, and XAS).

## 2. Results and Discussion

### 2.1. DRIFTS

**Figure 1:**
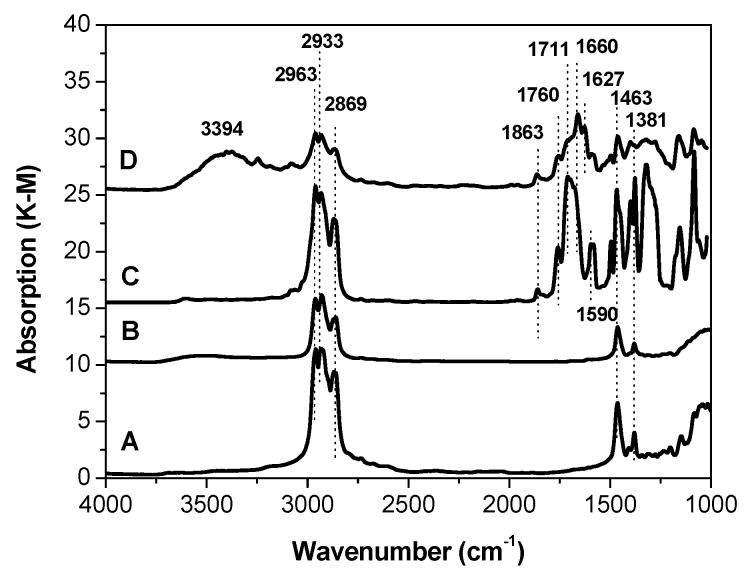
DRIFT survey spectra of propylene polymerization catalysts and carrier.

Figure 1 presents the DRIFT spectra of the starting samples after evacuation and before the adsorption of probe molecules. The spectra of A carrier and B catalyst contain absorption bands assigned to the alkyl fragments, presumably formed at the stage of MgCl_2_ synthesis. The bands at 2963–2869 cm^−1^ can be attributed to C–H stretching vibrations in CH_2_ group, whereas the bands at 1463–1381 cm^−1^ belong to the bending vibrations of the CH_2_ and CH_3_ groups [21].

The IR spectra of the C and D catalysts are more complicated: they contain bands characterizing the presence of absorption due to the modification of the catalysts with internal donors and a reduction agent. In addition, in the spectrum of sample D, a broad absorption band centered at about 3400 cm^−1^ is observed [22]. This band may be assigned to the stretching vibrations of OH groups that are hydrogen-bonded with some ligands.

**Figure 2:**
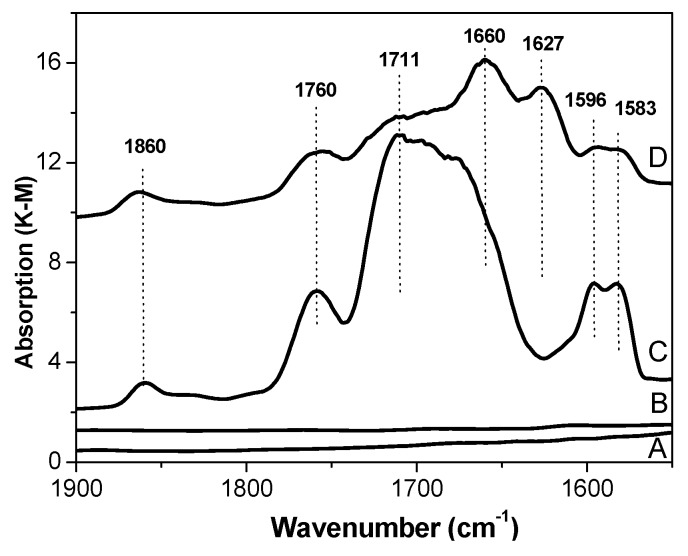
DRIFT spectra in the region of the carbonyl groups (internal donor) of the olefin polymerization catalysts and carrier.

OH groups, most probably caused by a hydrolysis of the reduction agent (Al(C_2_H_5_)_2_Cl), are seen in sample D exclusively. The absorption band at 1627 cm^−1^ can be assigned to the complex of (Al(C_2_H_5_)_2_Cl) with carboxyl group of DEP [23]. In contrast to support (A) and catalyst (B) in IR spectra of C and D catalysts (Figure 2), a few absorption bands belonging to stretching vibrations of carbonyl groups were observed [24]. The doublet band at 1596–1583 cm^−1^ most probably belongs to (TiCl_4_DEP)_2_ and (TiCl_4_DEHP)_2_ surface complexes. The wide band near 1700 cm^−1^ may be assigned to MgCl_2_DEP and MgCl_2_DEHP, whereas the band at 1660 cm^−1^ may be assigned to AlEt_2_Cl/DEHP surface complexes. The bands at 1760 and 1860 cm^−1^ observed in spectra of catalysts C and D are seemingly characteristic of physisorbed DEP and DEHP and/or their complexes with MgCl_2_, as proposed in the literature [25].

The CO adsorption at room temperature produces no changes in the spectra. This effect was observed for all the samples under study and indicates that strong low-coordinated Lewis acid sites capable of adsorbing a weak base like the CO molecule are not present at the surface of the catalysts and carrier. Heating of the samples at 85 °C in the presence of CO did not produce any changes in the IR spectra either. 

**Figure 3:**
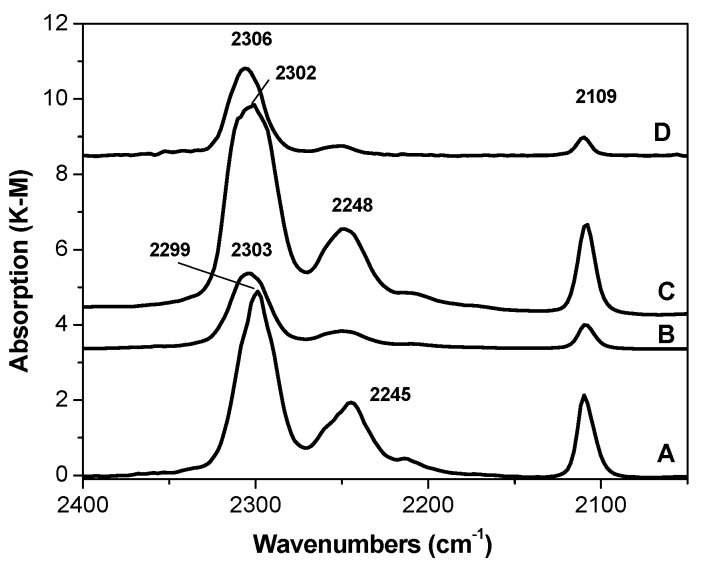
DRIFT spectra of CD_3_CN adsorbed at RT on the carrier and polymerization catalysts.

The IR spectra of the catalysts measured after adsorption of d_3_-acetonitrile are shown in Figure 3. It is seen that the adsorption of a stronger base (acetonitrile compared to CO) leads to the appearance in the spectra of all the samples of the absorption bands characteristic for С≡N stretching vibrations (2299–2306 cm^−1^) due to the formation of complexes with the so-called “shielded” Lewis acid sites (inaccessible to weak bases but accessible to strong bases due to the possibility of the latter changing the coordination sphere of the metal ions) [26,27,28]. In addition, the absorption bands attributed to unperturbed (physical adsorption) С≡N and С–D stretching vibrations (2248–2245 сm^−1^and 2109 сm^−1^, respectively) are observed. 

Comparison of the spectra of adsorbed d_3_-acetonitrile shows that stronger Lewis acid sites are observed for sample D. The shift of the frequency of the C≡N stretching vibrations for these catalysts is 53 cm^−1^ relative to the gas phase frequency (2253 cm^−1^). Somewhat weaker Lewis acid sites are present in the B and C catalysts, with the corresponding shifts being equal to 49–50 cm^−1^. The weakest Lewis acid sites (only Mg^2+^ cations) were found at the surface of the carrier (sample A). The С≡N frequency shift for this sample is 46 cm^−1^. It should be noted that sample C is most likely characterized by a rather broad distribution of the Lewis acid sites of different strengths, since a broadened absorption band is observed in the IR spectrum of this catalyst in the region of the C≡N stretching vibrations. The Lewis acid sites in the B, C, and D samples are Mg^2+^ together with Ti*^n^*^+^ (*n* = 4, 3, 2) cations. 

**Figure 4:**
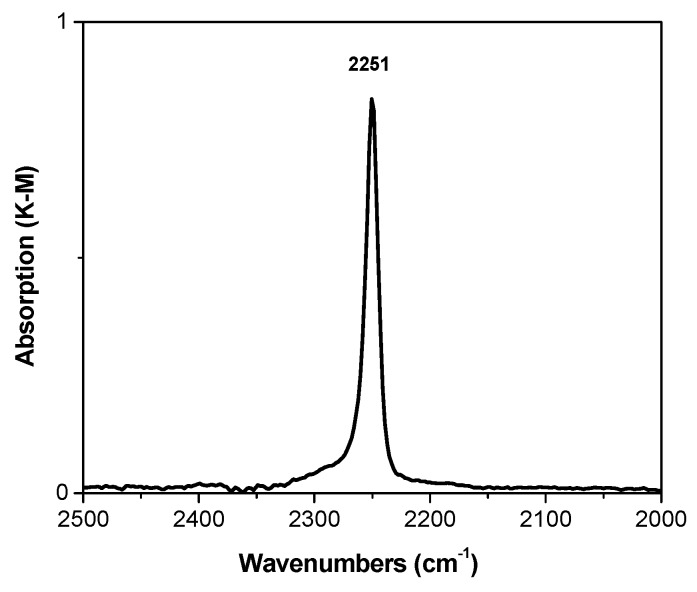
DRIFT spectrum of adsorbed CCl_3_D on D catalyst.

Figure 4 shows the spectrum of sample D, measured after adsorption of CDCl_3_ at room temperature. The spectrum contains only one band attributed to the stretching vibrations of the C–D bond at 2251 сm^−1^. This band is shifted by some 12 cm^−1^ to lower frequencies as compared to the CDCl_3_ frequency in the gas phase (2263 cm^−1^) [29]. From these data, it follows that no strong basic sites exist in this propylene polymerization catalyst; only moderate-strength sites are present in a significant concentration.

### 2.2. XPS 

**Figure 5:**
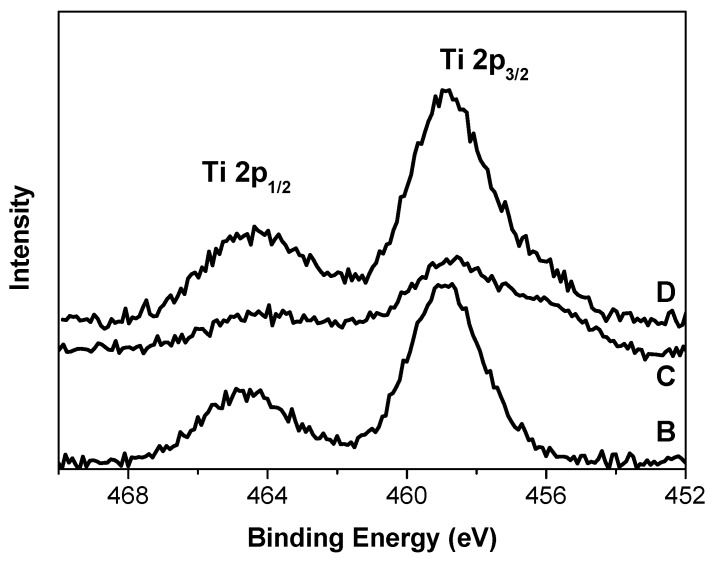
XP spectra of Ti 2p for the propylene polymerization catalysts.

X-ray photoelectron spectra of the Ti 2p region observed for the B, C, and D catalysts are shown in Figure 5. In Table 1, the positions and full widths at half maximum (FWHM) of the XPS peaks are collected, whereas Table 2 summarizes the surface atomic ratios. 

XPS survey spectra show the presence of O, C, Mg, Cl, Ti, and Al atoms in the surface layers of the B, C, and D catalysts. The electronic state of oxygen in the carrier (A) and catalysts (B and C) is about the same (Е_b_ O 1s = 532.4–532.7 eV). The FWHM of XPS peaks is almost identical (3.1–3.3 eV) (Table 1). It should be noted that the O 1s line in the XP spectra of sample D is broadened compared to sample C (3.6 vs. 3.2 eV) and Е_b_ O 1s is lower (531.8 vs. 532.4 eV). This fact can be accounted for by the presence of different oxygen-containing internal donors at the surface of these two catalysts and the presence of water in sample D (see Figure 1 and Figure 2).

The electronic state of chlorine is about the same in samples A and B (Е_b_ Cl 2p = 199.7–199.8 eV). A somewhat higher electron density on the chlorine atoms was found in samples C and D (Е_b_ Cl 2p = 199.2–199.5 eV). The FWHM of the Cl 2p line is the smallest for sample B (3.2 eV), whereas for the other samples it was 3.4 eV. 

**Figure 6:**
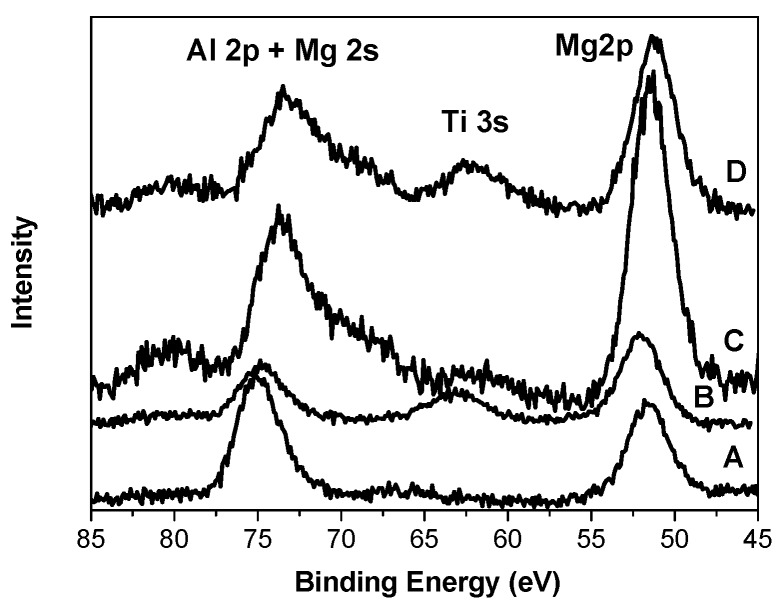
XP spectra of the Al 2p-Mg 2s-Ti 3s-Mg 2p electron levels in catalysts and carrier.

Figure 6 demonstrates XP spectra in the region of Al 2p, Mg 2s, Ti 3s, and Mg 2p electrons. The XPS lines of the Al 2p and Mg 2s electrons overlap. The presence of titanium compounds in sample B leads to a change in the state of magnesium and aluminum compared with the carrier (sample A) containing these two components only. The binding energy of Mg 2p electrons increases, whereas that of Al 2p electrons decreases (Table 1). The presence of the internal donors in two other samples results in an electron density increase on Mg in the C and D samples (Е_b_ Mg 2p = 51.4–51.2 eV). The binding energy of Al 2p electrons in both C and D samples decreases significantly as well. In addition, the Al 2p line in the XP spectra of samples C and D is wide (3.6–4.0 eV), while the width of the Mg 2p line is nearly the same (2.8–3.0 eV).

It is seen in Figure 5 that the Ti 2p line in the XP spectrum of sample B represents a narrow symmetric doublet with symmetrical components. On the contrary, the components of the Ti 2p doublet in the spectra of two other samples are broadened. The Ti 2p spectrum of sample C explicitly demonstrates the additional inhomogeneity as shoulders in the region of lower binding energies. From the position of the maxima of the Ti 2p_3/2_ line in the spectra of the catalysts and the width of these lines we assume the presence of one or several states of titanium in the surface layers of the catalysts available for XPS analysis. In the literature, there are no significant differences in the binding energies (B.E.) of the Ti 2p_3/2_ lines in the XP spectra of complexes of Ti^4+^ (or Ti^3+^) and solid compounds containing Ti^4+^ (or Ti^3+^) [8,10,30,31,32]. The position and shape of the Ti 2p_3/2_ line in the XP spectrum of sample B (E_b_ = 458.9 eV) and its FWHM (2.3 eV) (Table 2) show that both Ti states may be possible. Further differentiation by ESR study (see below) allowed us to identify the state of titanium in this sample as Ti^3+^. The mathematical treatment of Ti 2p spectra of samples C and D (Figures S1 and S2, supporting information) shows that titanium in this oxidation state is also present at the surface layers of C (about 60%) and D (about 80%) samples. The rest of titanium in the C and D samples is identified as a Ti species with a lower positive charge compared to sample B. Part of titanium can be identified as Ti^2+^ according to [33]. In addition, the Ti centers in sample C have a large electron density. The higher B.E. of the Ti 2p_3/2_ lines found in the XP spectra of sample D might be caused, according to [9], by the presence of H_2_O in this sample. The presence of water at the surface of this sample was confirmed by DRIFT (see Figure 1). 

Table 2 summarizes the atomic ratios of the components with respect to magnesium, which is present in all these samples. There is some chlorine deficit at the surface of the starting MgCl_2_ sample (the Cl/Mg ratio is equal to 0.78 instead of 2). It is seen that the atomic ratio Ti/Mg is higher in the surface layers of the MgCl_2_ + TiCl_4_ sample (B) as compared to the other samples under study. 

### 2.3. ESR 

**Figure 7:**
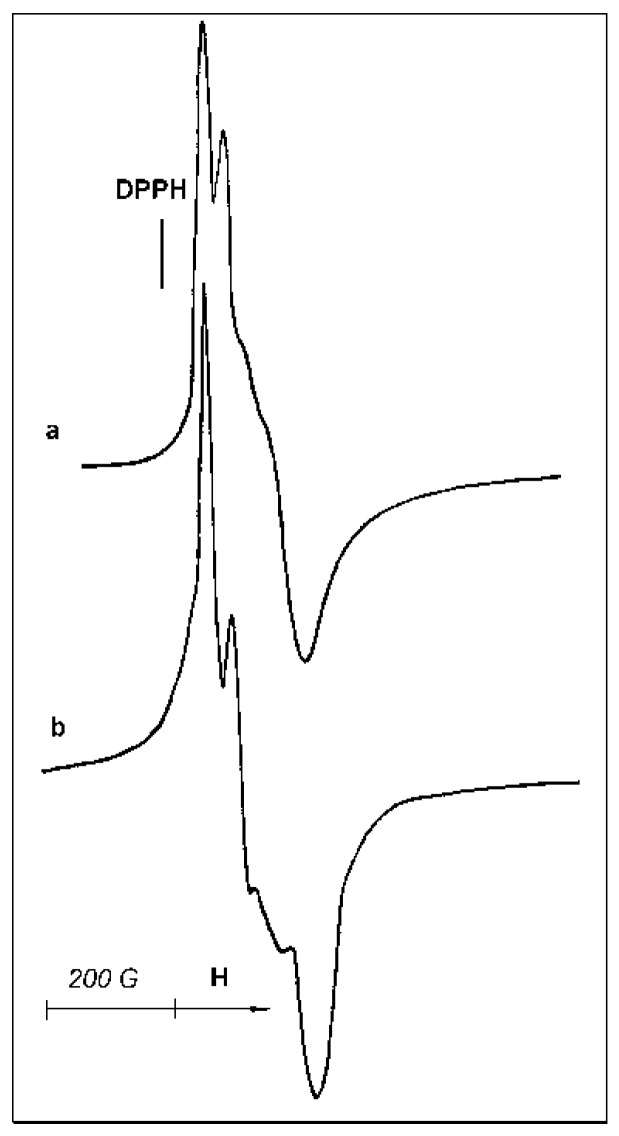
ESR spectra, taken at 20 °C, of sample B: (**a**) initial; (**b**) after CO adsorption at 1.3 kPa.

In ESR testing of samples, paramagnetic Ti^3+^ ions located both on the surface and in the volume of catalyst particles are detected. However, peculiarities of sample reoxidation or interactions with ligand molecules allow us to draw some conclusions about the surface/bulk location of paramagnetic sites. The starting sample B demonstrates a rather strong Ti^3+^ ESR signal at room temperature (Figure 7a). CO adsorption causes only a minor change in the signal shape (Figure 7b). 

**Figure 8:**
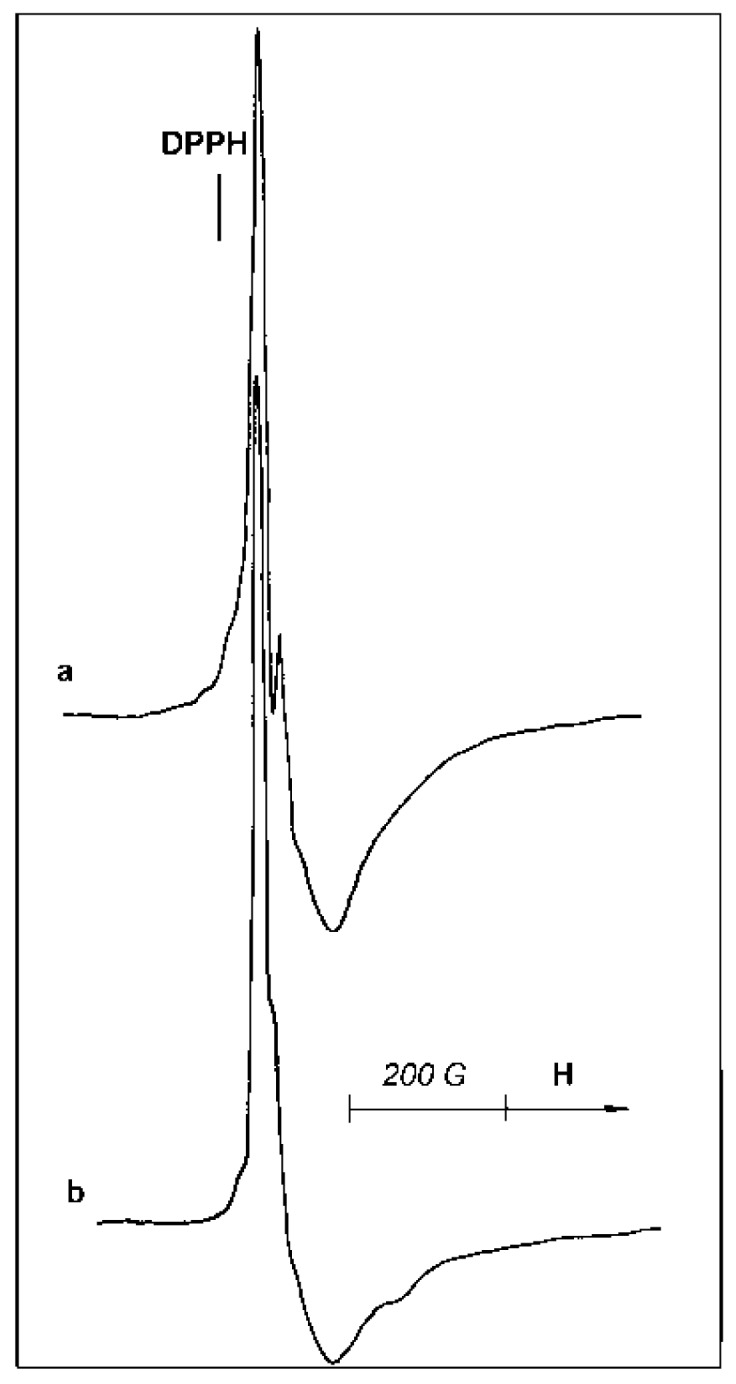
ESR spectra, taken at 20 °C, of sample B: (**a**) with acetonitrile adsorption at 12 kPa; (**b**) after evacuation at 50 °C for 30 min.

Acetonitrile adsorption on this catalyst is accompanied by more pronounced changes in the signal shape and asymmetry (Figure 8a), demonstrating a measurable transformation of the paramagnetic site symmetry. Subsequent evacuation of the sample at 50 °C does not lead to the restoration of the parent ESR signal (Figure 7a) but causes only minor changes of the signal shape (Figure 8b), confirming that acetonitrile molecules are strongly bonded in the complex formed.

Interaction of sample B with air is accompanied by the appearance of a very weak narrow ESR signal at g = 2.003, indicative of the formation of a small amount of O_2_^−^ species. However, the low intensity of this signal casts into doubt the relationship between the newly formed O_2_^−^ species and the existing Ti^3+^ paramagnetic sites. Moreover, the exposure of sample B to air does not cause an immediate disappearance of the ESR signal of paramagnetic Ti-sites; rather, it begins a slow decay of the signal (Figure 7a) that lasts for hours. Therefore, a considerable portion of ESR-visible Ti^3+^-ions seems to be located inside the bulk of the active phase. 

**Figure 9:**
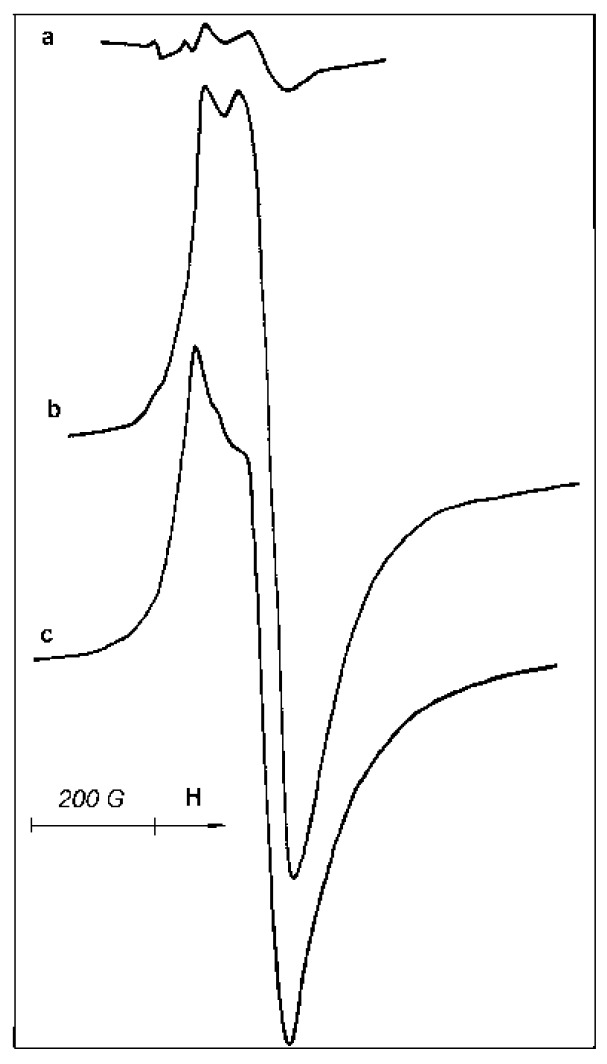
ESR spectra, taken at 20 °C, of samples C (**a**) and D: (**b**) initial; (**c**) after acetonitrile adsorption at 12 kPa.

Two ESR signals in samples C and D are presented in Figure 9a,b, respectively. Comparison of these signals, taken at identical magnifications from two identical probes, illustrates a sharp difference in the concentration of paramagnetic sites in these catalysts.

No changes in the ESR signal (Figure 9b) take place as a result of CO adsorption on sample D. Rather insignificant irreversible transformation of the signal shape is observed after acetonitrile adsorption (Figure 9c). Surprisingly, the catalyst is very resistant to air exposure at 20 °C: after 16 h of exposure to air, the ESR signal (Figure 9b) of paramagnetic Ti-sites retains ~75% of the original intensity. Once more, a main part of paramagnetic Ti^3+^-sites detected in sample D by ESR is located in hindered positions inside catalyst particles and takes no part in the interaction with gas phase molecules.

**Figure 10:**
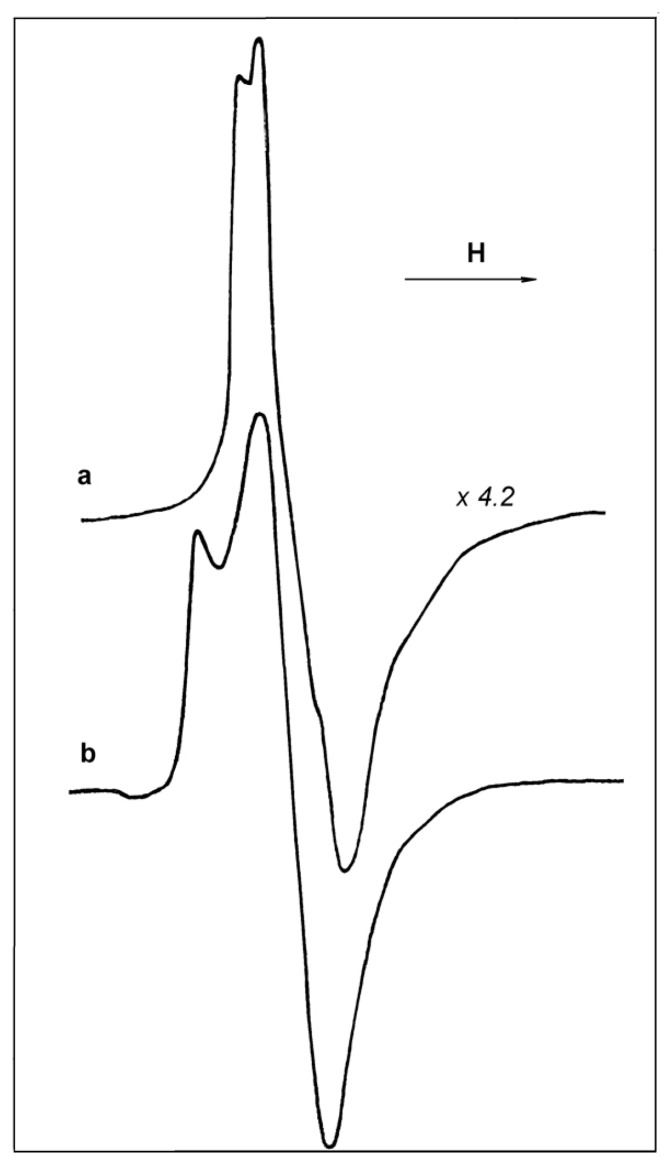
ESR spectra, taken at −196 °C, of the initial samples B (**a**) and D (**b**).

Two ESR signals, taken at −196 °C, for samples B and D are presented in Figure 10a,b. It is clearly seen that the signals differ in their shape. Thus, the catalysts differ in the coordination/structure of the paramagnetic sites. Double integration of the two spectra permits us to conclude that the number of paramagnetic sites in sample D is maximal and exceeds the concentration of such centers in sample B by a factor of ~3.5.

**Figure 11:**
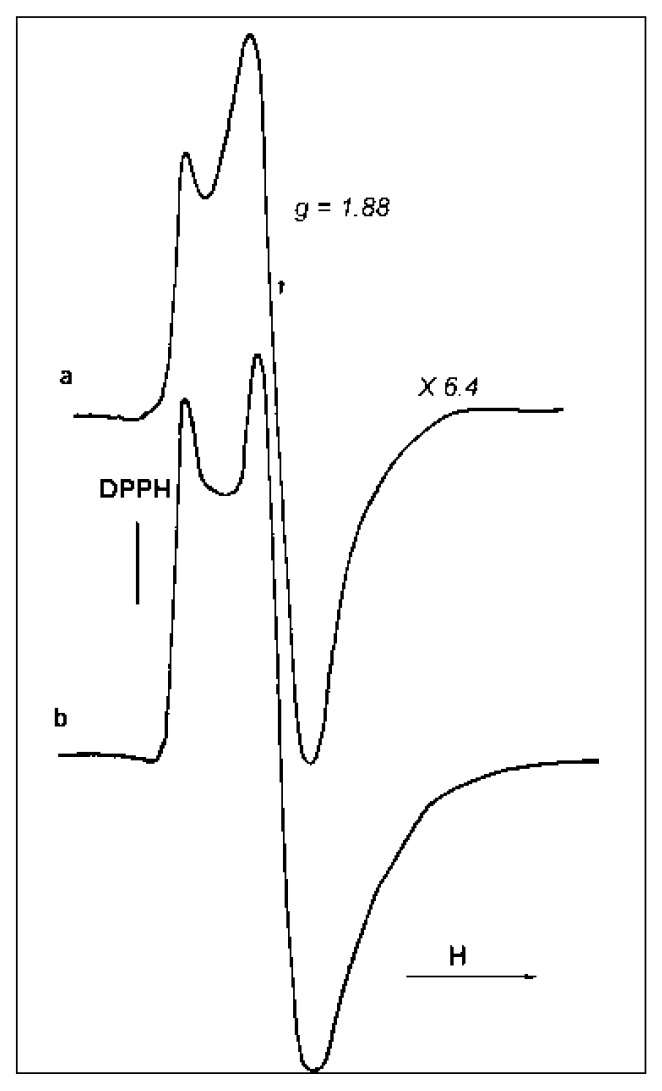
ESR spectra, taken at −196 *°*C, of sample D (**a**) and a frozen solution of 8.5 wt % TiCl_3_ in concentrated HCl (**b**).

The ESR spectrum of sample D is compared with a standard (frozen TiCl_3_ solution) taken as a reference for the evaluation of the number of “ESR-visible” Ti^3+^-sites in the samples (Figure 11). According to this evaluation, the number of paramagnetic centers in the catalyst D reaches 1–1.5% (wt % of Ti). Taking into account that the total Ti concentration in the samples averages 2–4 wt %, one can conclude that the considerable part (~50%) of Ti in D catalyst forms isolated paramagnetic sites. From the identity of the signal shapes, one can suppose that paramagnetic sites in this catalyst resemble isolated Ti^3+^ species in the frozen TiCl_3_ solution. 

### 2.4. XAS

**Figure 12:**
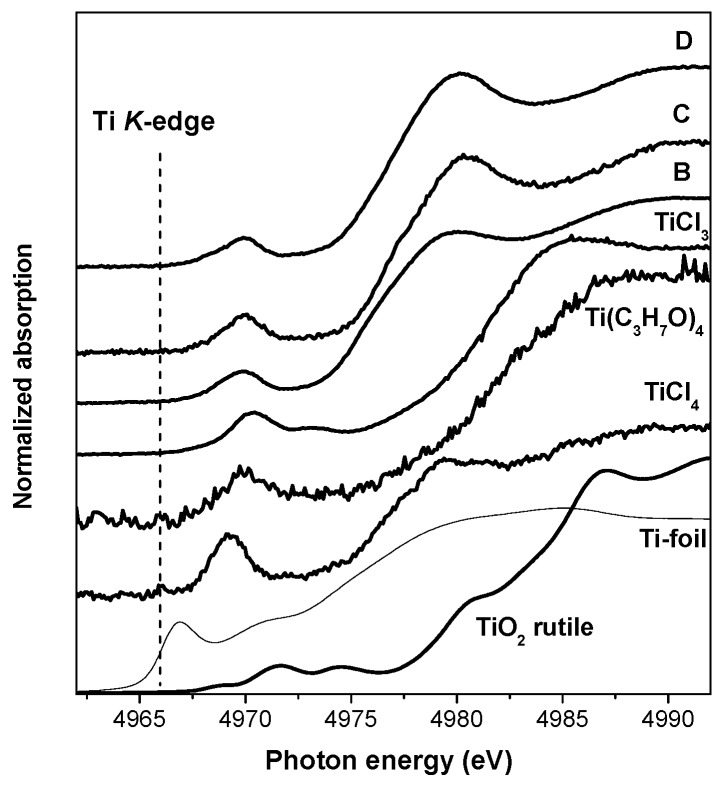
Normalized XANES spectra of the Ziegler–Natta polymerization catalysts and references.

The Ti *K*-edge normalized XANES spectra of the catalysts and the reference samples are depicted in Figure 12. It is known that the pre-edge features of transition metals are related to the coordination number, oxidation state of adsorbing atom, and symmetry of the adsorbing atom site [34,35,36]. The pre-edge features in the spectra of the Ziegler–Natta catalysts are similar to each other, but they differ from those of reference compounds: Ti-foil, TiO_2_ rutile, TiCl_3_, TiCl_4_, and Ti(C_3_H_7_O)_4_. There is one narrow peak in the spectra of the Ziegler–Natta polymerization catalysts, in contrast to three of them in the TiO_2_ spectrum. There is one pre-edge peak in the spectrum of TiCl_4_ as well, but the pre-peak normalized height of the samples under study is around 0.25, whereas the normalized height of the pre-peak for TiCl_4_ is about 0.55 (Figure S3, Supplementary Materials). Moreover, the pre-edge peak in the TiCl_4_ spectrum is located at a lower energy (0.6 eV) compared with that in the spectra of the Ziegler–Natta catalysts under study. There are two pre-edge peaks in the spectrum of TiCl_3_ and the position of the main peak is shifted to a higher energy (0.5 eV). There is one pre-edge peak in the spectrum of tetrabutoxy titanium located at the same energy as in the spectra of the Ziegler–Natta catalysts. Thus, the fingerprint approach indicates the absence of free TiCl_4_ tetrahedral clusters in the Ziegler–Natta polymerization catalysts. It indicates that the Ti species in these catalysts are definitely not Ti^4+^ ions surrounded by six oxygen atoms and not Ti^3+^ ions surrounded by three chlorine atoms. 

The position of the Ti *K*-edge in the spectra of the polymerization catalysts is lower than that in TiO_2_, TiCl_3_, and Ti(C_3_H_7_O)_4_ but similar to that in the spectrum of TiCl_4_. However, the shape of XANES and the height of the white line in the spectra of samples C and D differ from TiCl_4_. The spectrum of sample B differs from TiCl_4_ in the position and height of the pre-edge peak. This means that Ti in the studied Ziegler–Natta polymerization catalysts, perhaps, exists as a mixture of Ti^4+^, Ti^3+^, Ti^2+^, and/or Ti^1+^ electronic states. These results are in good agreement with the conclusions drawn from the XPS data (see above). 

Furthermore, one can deduce by analyzing the position of the Ti *K*-edge and the intensity of the white line that the Ti species in samples C and D have, on average, a more electron-deficient character (surrounded by more electron-accepting neighbors), whereas sample B demonstrates a lower electron-deficient character (more electron-donating neighbors). The difference in the edge position can indicate the difference in the bond ionicity of Ti with nearest neighbors as well. In this case, the average bond ionicity is higher in the case of samples C and D and is lower in sample B. 

**Figure 13:**
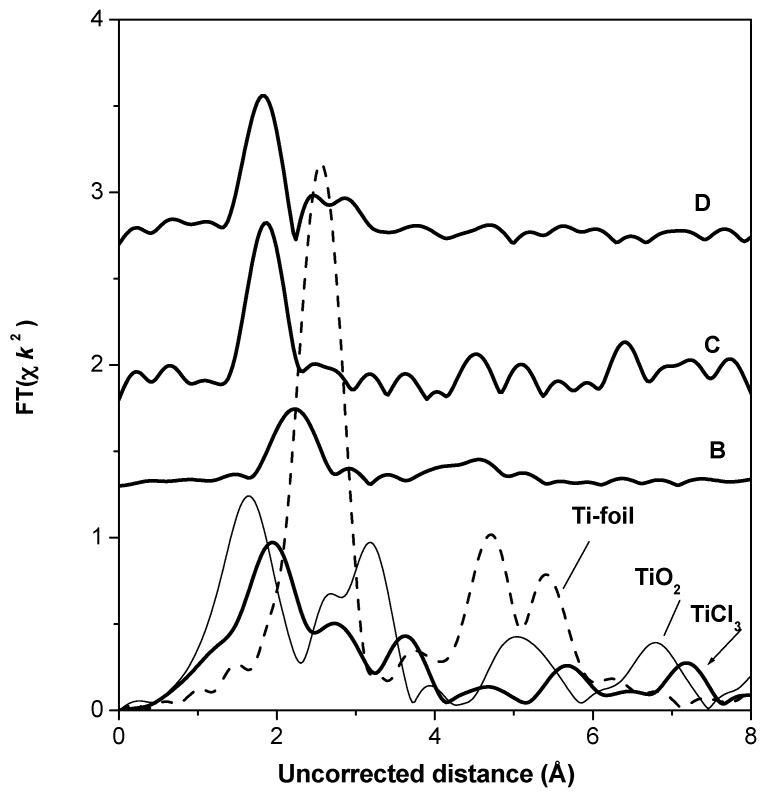
Fourier transformations of the EXAFS spectra of the Ziegler–Natta polymerization catalysts and references.

The Fourier transformations of XAS oscillations for the Ziegler–Natta polymerization catalysts and references are presented in Figure 13. EXAFS oscillation of Ti(C_3_H_7_O)_4_ reference compound is too noisy; therefore, FT is uncertain and is not shown. One peak is observed in the spectra of the B, C, and D catalysts. The position of this peak is near 2.2 Å uncorrected distance in the spectrum of sample B, whereas it is near 1.8 Å in the spectrum of the other two catalysts.

In the spectrum of TiO_2_, the first peak corresponds to the coordination shells containing O atoms at about 1.6 Å uncorrected distance, whereas in the spectrum of TiCl_3_ the first peak corresponds to the coordination shell containing Cl atoms. 

The position of the first peak in the spectra of catalysts shows the possibility of the Ti–Cl atomic pair presence in the first coordination shell. The first peak of the Ti FT EXAFS spectra was fitted in both r- and k-spaces with a one-shell model—a chlorine shell around the central absorbing titanium atom (Figure S4, Supplementary Materials). The results of the model fit are presented in Table 3. It is seen that the first shell contains one Cl atom at the real distance 2.25 Å in sample C. The real distance in the Ti–Cl atomic pair in sample D is considerably shorter (2.23 Å), than in sample B (2.54 Å). 

## 3. Materials and Methods

Four samples: A—MgCl_2_ containing catalyst support; B—MgCl_2_-based TiCl_4_ catalyst of ethylene polymerization; С and D—MgCl_2_-based TiCl_4_ catalysts with the internal donor for propylene polymerization, were studied (Table 4).

Diffuse-reflectance Fourier-transform infrared spectra of the polymerization catalysts were recorded at room temperature using a NICOLET “Protege” 460 spectrometer with a homemade diffuse-reflectance unit [40]. To obtain a satisfactory signal-to-noise ratio, 200 scans were collected per spectrum. The spectra were measured from 400 to 4000 cm^−1^ with a resolution of 4 cm^−1^. The powdered samples of the catalysts were placed in the ampoules using a glove box under a dried Ar atmosphere. The same quartz ampoules were used for ESR measurements. The probe molecules CO (1.3 kPa), CD_3_CN (12 kPa), and CCl_3_D (18.7 kPa), were adsorbed at room temperature. Before adsorption, non-activated samples were treated in a vacuum (0.13 Pa) for 20 min at ambient temperature.

Carbon monoxide and d_3_-acetonitrile were used as probe molecules for Lewis acid sites because they differ in their basicity and can differentiate between “exposed” Lewis acid sites, i.e., the sites with a low-coordinated cation exposing its electron vacancy for the interaction (like trigonal sites), and “shielded” Lewis acid sites, i.e., sites with a pseudo-saturated coordination sphere. In the latter case, a probe-molecule like CD_3_CN is capable of withdrawing the cation from its coordination sphere and increasing its coordination number. For instance, when we have a MO_4_ tetrahedron in which the metal ion is shielded by four oxygen atoms, adsorption of a strong ligand (probe molecule) may result in the insertion of the fifth ligand into the coordination sphere of the metal [41]. In addition to probe molecules for acid sites and low-coordinated ions, we also used a probe molecule for basic sites, i.e., chlorine atoms of the metal chlorides forming the support and the catalyst, as well as oxygen atoms in the composition of the organic electron-donor modifiers used to activate the catalyst. For this purpose, the molecule of deuterated chloroform, as a weak acid, was chosen because of the presence of intense CH bonds on the initial catalyst surface before the adsorption of the probe molecule and thus the CH-containing probes cannot be used for this purpose. Although the background in the region of C–D vibrations was not good for reliable spectra measurement, we succeeded in collecting some data about the basic sites at the surface of the polymerization catalysts. Up to now we studied only the adsorption of CHCl_3_ on various solid carriers and catalysts and some representative shifts reflecting the measure of basicity due to the surface ^…^O^…^H–CCl_3_ complexes are collected in Table 5. Obviously, the stronger the O^…^H interaction in this complex, the larger the red shift of the C–H stretching vibration, and vice versa. The corresponding shifts in the C–D region are approximately equal to the value of the C–H shift divided by √2 [42]. For sample D the spectrum was also taken after heating in СО at 85 °С for 10 min. For the same sample, adsorption of CDCl_3_ at room temperature was carried out.

XPS spectra were recorded using a XSAM 800 spectrometer with Mg Kα X-ray (1253.6 eV) source. The base pressure in the XPS chamber was about 1.3 × 10^−5^ Pa. The spectrometer was calibrated using the binding energy (E_b_) of the Au 4f_7/2_ level = 84.0 eV and Ni 2p_3/2_ = 852.7 eV. Survey spectra were collected between 20 and 1130 eV. Detailed spectra were recorded for the region of C 1s, O 1s, Mg 2p, Cl 2p, Al 2p, and Ti 2p with a 0.1 eV step. The C 1s line at 285.0 eV was used for energy calibration. The Mg 2p line (51.6 eV) was used as an internal standard for the carrier. The surface atomic composition and atomic ratio were calculated after a Shirley type background subtraction [43] by dividing the peak area by the photo-ionization cross-sections [44]. The experimental Ti 2p spectra have been deconvoluted in a series of mixed Gaussian–Lorentzian shape contributions. 

ESR spectra were taken in the X-band (λ ≅ 3.2 cm) at 20 °C and −196 °C using a reflecting-type ESR spectrometer equipped with a 4104OR cavity and quartz Dewar vessel. The ESR signals were registered in the absence of saturation in the field range of 2000–4000 G. The Excel program was used for spectra processing (baseline correction, double integration). DPPH and a frozen solution of 8.5 wt % TiCl_3_ in concentrated HCl were used as standards for g-factor calculation and quantitative Ti^3+^ ESR analysis, respectively.

The samples of catalysts were inserted into identical quartz ampoules in a glove box for combined use in ESR study and DRIFT spectroscopy. The amount of the sample in the cylindrical ESR part of the ampoule (3.5 mm in diameter) exceeded the volume of the sample in the resonator (>30 mm height). ESR spectra were registered at 20 °C and −196 °C immediately after the DRIFT measurements. Next, closed ampoules were connected to the adsorption setup and the samples were treated with CO or d_3_-acetonitrile. Then DRIFT and ESR measurements at 20 °C and −196 °C were repeated. The solution of 8.5 wt % TiCl_3_ in (30% HCl + H_2_O) was used as a standard for the comparative evaluation of the “ESR-visible” fraction of Ti^3+^ in the samples. Double integration of the ESR spectrum of this standard provided the absolute value for comparison with double integrals of the signals under study. The shape of the ESR lines does not play any role, and the spectra simulation was not required. For the sake of accuracy, the series of the samples were measured consecutively, with ampoules placed in the same position inside the ESR resonator one after another. 

X-ray absorption spectra (Ti *K*-edge at 4966 eV) were measured at HASYLAB E4 station (DESY, Hamburg, Germany). The X-ray beam was monochromatized with Si(111) double crystal detuned to 50% of the maximum intensity to avoid higher harmonics contamination of the monochromatized beam. The spectra μ(E) were measured at −196 °C in a transmission mode using ionization chambers. The spectrum of Ti metal foil (between the second and third ionization chambers) was recorded at the same time for energy calibration purposes.

Special care was taken to avoid air contamination. For sample preparation, a mixture of about 10 mg of the catalyst (the optimum weight to maximize the signal-to-noise ratio) and 20 mg of polyethylene powder (spectrophotometric grade, Aldrich) as a binding agent was pressed into a 13-mm diameter pellet in a glove box (a dried Ar atmosphere) and completely wrapped in Kapton polyimide tape with silicone adhesive. The aim of dilution was to provide transmission of approximately 30% of the beam. However, a good signal to noise ratio was not always achieved, because a significant portion of the beam absorption came from other atoms, such as Mg, Al, Si, and Cl. Reference spectra for Ti^0^ and Ti^4+^ oxidation states were recorded using standard reference samples (Ti-foil, TiO_2_ (rutile), TiCl_3_, TiCl_4_, and Ti(C_3_H_7_O)_4_). 

Data treatment was carried out with the software package VIPER [45]. In the spectra of the absorption coefficient μ, a Victorian polynomial was fitted to the pre-edge region for background subtraction. A smooth atomic background, μ_0_(*k*), was evaluated using smoothed cubic splines. The radial distribution function FT[*k*^2^χ(*k*)] was obtained by Fourier transformation of *k*^2^-weighted experimental function χ = (μ(*k*) − μ_0_(*k*))/μ_0_(*k*) performed with a Bassel window. For the determination of structural parameters, theoretical references calculated by the FEFF8.10 code were used [46]. Duplicate spectra were recorded to ensure data reproducibility.

## 4. Conclusions

We characterized a series of Ziegler–Natta polymerization catalysts by a complex of surface- and bulk-sensitive physical methods. DRIFTS study showed the presence of rather strong Lewis acid sites able to interact only with rather strong bases (acetonitrile) but not with weak molecules (CO). There are no strong basic sites in the polymerization catalysts; instead, only moderate-strength sites are present in a significant concentration. XPS analysis revealed the presence of Ti^3+^ and Ti^1+^ species in the surface layer of catalysts, with different ratios ranging from 100% of the Ti^3+^ species (sample B) and 60% (sample C) to 80% (sample D). The total concentration of Ti in the surface layers diminishes in the following order: B > D > C. The samples under study differ drastically in terms of the total number (surface + bulk) of ESR-visible paramagnetic sites. The concentration of paramagnetic Ti^3+^-centers in the sample volume drops from ~2 wt % Ti to 0 in the order D > B >> C. The XANES study confirms the presence of a mixture of Ti^4+^, Ti^3+^, Ti^2+^, and Ti^1+^ electronic states of Ti in the bulk of the catalysts under study. The EXAFS study shows the presence of a Cl atom as the nearest neighbor of the absorbing Ti atom. Here the local structure (the Ti–Cl distance and coordination number) of samples B, C, and D differs.

## Figures and Tables

**Table 1:** XPS data of propylene polymerization catalysts and support.

	Binding Energy (FWHM) (eV)				
	O 1s	Cl 2p	Mg 2p	Al 2p	Ti 2p_3/2_
A	532.7 (3.1)	199.8 (3.4)	51.6 (2.8)	75.0 (3.1)	-
B	532.4 (3.3)	199.7 (3.2)	52.0 (3.0)	74.8 (3.3)	458.9 (2.3) (Ti^4+,3+^)
C	532.4 (3.2)	199.5 (3.4)	51.4 (2.8)	73.6 (3.6)	458.7 (2.3)—60% (Ti^4+,3+^) 456.0 (2.3)—40% (Ti^2+,1+^)
D	531.8 (3.6)	199.2 (3.4)	51.2 (2.8)	73.1 (4.0)	458.9 (2.2)—80% (Ti^4+,3+^) 456.3 (2.2)—20% (Ti^2+,1+^)

**Table 2:** XPS surface atomic composition.

Sample	Atomic Ratio		
	Cl/Mg	Al/Mg	Ti/Mg
A	0.78	0.75	-
B	0.90	0.32	0.33
C	1.02	0.22	0.06
D	1.02	0.49	0.23

**Table 3:** EXAFS data for the Ziegler–Natta polymerization catalysts.

Sample	Pair	R (Å)	CN	σ (Å^2^)	∆E (eV)
B	Ti–Cl	2.539 ± 0.006	0.6 ± 0.1	0.006 ± 0.001	18.7 ± 0.5
C	Ti–Cl	2.253 ± 0.005	1.3 ± 0.1	0.008 ± 0.001	18.4 ± 0.5
D	Ti–Cl	2.229 ± 0.008	0.7 ± 0.1	0.005 ± 0.001	10.4 ± 0.1

**Table 4:** Catalyst and catalyst carrier samples.

Preparation Method		Bulk Content (wt %)		
		Ti	Al	Mg
A	MgCl_2_ containing carrier material, prepared from magnesium alkoxide by reacting it with ethylaluminium dichloride. Magnesium alkoxide was prepared by reacting magnesium alkyl with a stoichiometric amount of alcohol. Carrier material was prepared according to example 3 in patent [37].	-	0.6	0.63
B	Catalyst was prepared from carrier sample A by reacting it with TiCl_4_ and washing with heptane as described in example 3 in patent [37].	8.0	1.3	12.7
C	Ziegler-Natta catalyst prepared as described in example 3 in patent [38] with diethyl phthalate (DEP) as an internal donor.	1.96	0.1–0.2	20.2
D	Ziegler-Natta catalyst prepared as described in patent [39] in example 5. Bis(2-ethylhexyl)phthalate (DEHP) was used as an internal donor and diethylaluminium chloride (Al(C_2_H_5_)_2_Cl) was used as a reduction agent.	4.2	0.1–0.2	13.1

**Table 5:** Representative frequency shifts after adsorption of CHCl_3_ on different solid materials [24].

Material	Frequency Shift (cm^−1^)
Na-mordenite (zeolite)	10
Na-Y zeolite	24
ZrO_2_	29
MgO	29, 64
Na-X zeolite	42
Cs_2_CO_3_/NaX zeolite	52
Polymerization catalysts	14–15 *

* Calculated from the C–D frequency.

## Supplementary Materials

The following supplementary materials are available online at www.mdpi.com/1996-1944/10/5/496/s1, Figure S1: Ti 2p XP spectrum deconvolution of sample C, Figure S2: Ti 2p XP spectrum deconvolution of sample D, Figure S3: Normalized pre-edge height vs. energy position for Ti K-pre-edge features, Figure S4: Model fits of Ti *K* EXAFS of sample B in k-space (a) and r-space (b).
