# Supplementary material for: A Study of Ziegler–Natta Propylene Polymerization Catalysts by Spectroscopic Methods
Source: Materials (Basel). 2017 May 3;10(5):496. doi: 10.3390/ma10050496 (PMC5459041; doi:10.3390/ma10050496)
Supplement: Supplementary file 1 [file materials-10-00496-s001.pdf]

A Study of Ziegler–Natta Propylene Polymerization Catalysts by Spectroscopic Methods

Olga P. Tkachenko, Alexey V. Kucherov, Leonid M. Kustov, Ville Virkkunen, Timo Leinonen and Peter Denifl

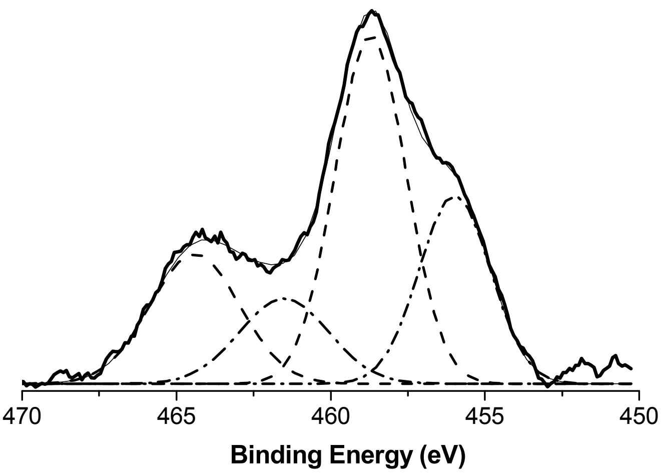

Figure S1. Deconvolution of XPS Ti 2p doublet of sample C.

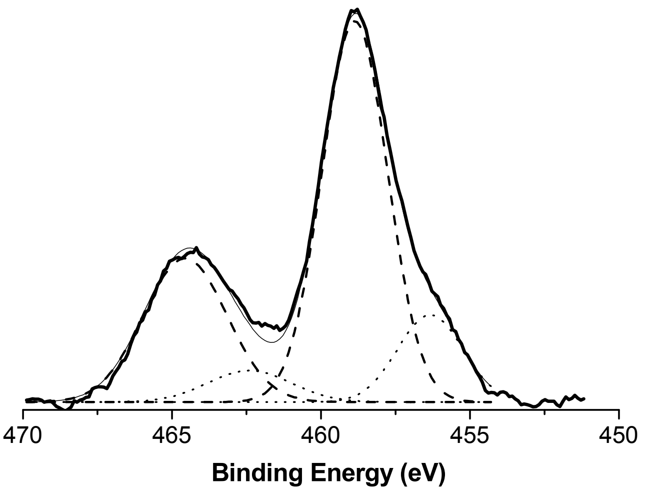

Figure S2. Deconvolution of XPS Ti 2p doublet of sample D.

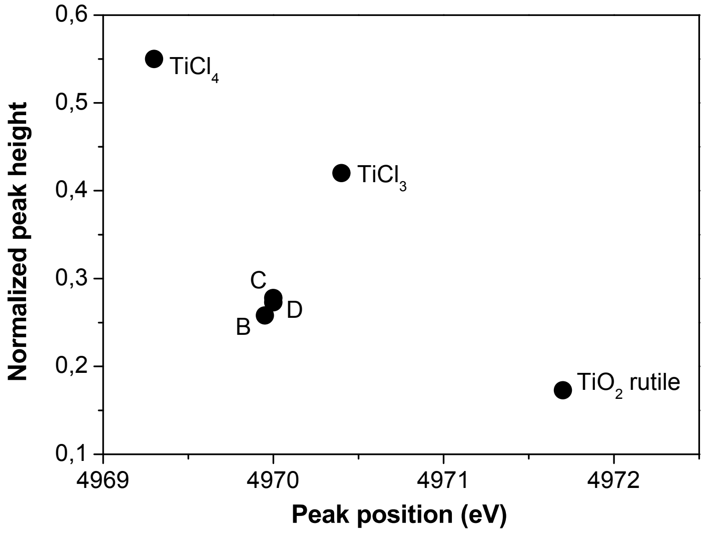

Figure S3. Normalized pre-edge height vs. energy position for Ti K-pre-edge features.

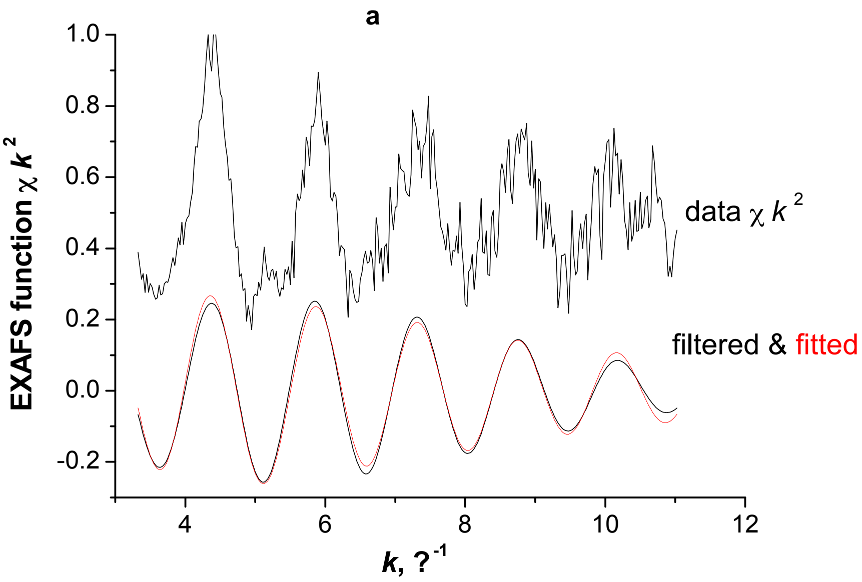

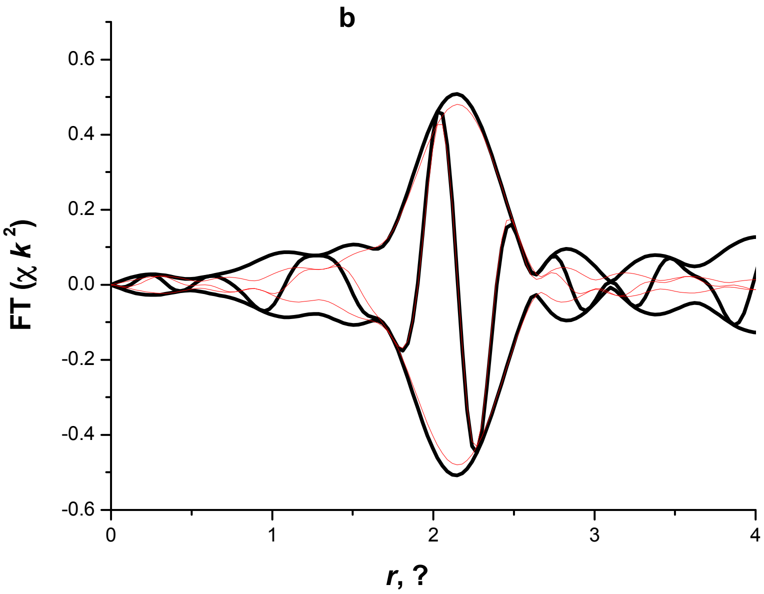

Figure S4. Model fits of the Ti *K*-edge EXAFS spectrum of sample B in k-space (**a**) and r-space (**b**).
